# Melioidosis after Hurricanes Irma and Maria, St. Thomas/St. John District, US Virgin Islands, October 2017

**DOI:** 10.3201/eid2510.180959

**Published:** 2019-10

**Authors:** Irene Guendel, Lisa LaPlace Ekpo, Mary K. Hinkle, Cosme J. Harrison, David D. Blaney, Jay E. Gee, Mindy G. Elrod, Sandra Boyd, Christopher A. Gulvik, Lindy Liu, Alex R. Hoffmaster, Brett R. Ellis, Tai Hunte-Ceasar, Esther M. Ellis

**Affiliations:** Virgin Islands Department of Health, St. Thomas, Virgin Islands, USA (I. Guendel, L. LaPlace Ekpo, C.J. Harrison, B.R. Ellis, T. Hunte-Ceasar, E.M. Ellis);; Walter Reed Army Institute of Research, Silver Spring, Maryland, USA (M.K. Hinkle);; Centers for Disease Control and Prevention, Atlanta, Georgia, USA (D.D. Blaney, J.E. Gee, M.G. Elrod, S. Boyd, C.A. Gulvik, L. Liu, A.R. Hoffmaster)

**Keywords:** melioidosis, Burkholderia pseudomallei, bacteria, natural disaster, hurricanes, Hurricane Irma, Hurricane Maria, antimicrobial resistance, public health, St. Thomas/St. John District, US Virgin Islands, United States

## Abstract

We report 2 cases of melioidosis in women with diabetes admitted to an emergency department in the US Virgin Islands during October 2017. These cases emerged after Hurricanes Irma and Maria and did not have a definitively identified source. Poor outcomes were observed when septicemia and pulmonary involvement were present.

Melioidosis is caused by *Burkholderia pseudomallei*, a saprophytic, gram-negative bacillus endemic to tropical regions worldwide (*1*). Diagnosis is difficult because of wide-ranging clinical manifestations (*2*), and this bacterium is innately resistant to many antimicrobial drugs, making treatment options limited, complex, and lengthy (*3*). Infection occurs by percutaneous exposure, inhalation, or ingestion.

Melioidosis is rare in the United States, and cases are usually travel related (*4**,**5*). However, regional endemicity has been documented in Puerto Rico (*6*), and sporadic human cases have been reported in the Caribbean (*5**,**7*). In September 2017, the US Virgin Islands were affected by 2 category 5 hurricanes, Irma and Maria; widespread flooding continued for weeks. We describe the clinical manifestations, management, and outcome of posthurricane melioidosis cases in 2 women in St. Thomas and St. John, US Virgin Islands.

## The Study

Despite major damage to the 2 hospitals in the territory during the 2 hurricanes, the Virgin Islands Department of Health (VIDOH) maintained surveillance at both emergency departments. Two isolates were recovered from each patient. Local specimen analysis for organism identification was performed by using the MicroScan WalkAway System (Siemens Healthcare Diagnostics, https://www.siemens-healthineers.com). All isolates were confirmed as *B. pseudomallei* at the Centers for Disease Control and Prevention (CDC; Atlanta, GA, USA). Whole-genome sequencing and single-nucleotide polymorphism analysis were performed (National Center for Biotechnology Information, https://www.ncbi.nlm.nih.gov, Project PRJNA488733). Genomes from a given patient were clonal to each other. However, representative genomes from both patients had differences (>5,600 single-nucleotide polymorphisms), indicating the presence of different strains in these infections. Genomic comparison with a reference panel indicated that the isolates were within the previously described Western Hemisphere clade and subclade associated with the Caribbean (*8*).

Patient 1 was an 80-year-old female resident of St. Thomas who had a history of cardiomyopathy and type II diabetes mellitus. She came to the emergency department (ED) at Schneider Regional Medical Center (St. Thomas, US Virgin Islands) because of shortness of breath (symptom onset 28 days after Hurricane Irma and 9 days after Hurricane Maria). Her symptoms were worsened orthopnea, increased abdominal girth, and edema, consistent with her symptoms at previous admissions. The patient was admitted for management of acute decompensated heart failure.

The patient had a temperature of 98.5°F; diffuse pulmonary crackles; jugular venous distension; normal heart sounds; and bilateral, lower extremity pitting edema. Examination showed a focal area on the anterior left thigh that had a central, firm, warm, erythematous, tender, subcutaneous nodule ≈2 cm in diameter with a central fluctuant area and a small pinhole. Incision and drainage was performed, and a swab specimen of purulent drainage was sent for culture.

The patient was given intravenous clindamycin (600 mg every 8 h for 5 d) and was discharged while receiving oral clindamycin, but the treatment course was not completed. Cultured wound showed growth of *B. pseudomallei* at ≈5 days. However, culture growth was not yet positive before patient discharge. The isolate was susceptible to trimethoprim/sulfamethoxazole (Table 1).

**Table 1:** Culture results and antimicrobial drug susceptibility for *Burkholderia pseudomallei* isolated from 2 case-patients with melioidosis after Hurricanes Irma and Maria, St. Thomas/St. John District, US Virgin Islands, October 2017*

Patient, culture	Drug	MicroScan Walk Away System MIC, µg/mL†	Result	CDC MIC, µg/mL	Result
1, first wound culture	Amoxicillin/clavulanate	NT	NA	4/2	S
	Ceftazidime	>16	R	4	S
	Doxycycline	NT	NA	1	S
	Imipenem	NT	NA	0.5	S
	Tetracycline	NT	NA	4	S
	Trimethoprim/sulfamethoxazole	<2/38	S	<0.5/9.5	S
	Meropenem	NT	NA	1	‡
1, second wound culture	Amoxicillin/clavulanate	NT	NA	4/2	S
	Ceftazidime	>16	R	2	S
	Doxycycline	NT	NA	1	S
	Imipenem	NT	NA	0.5	S
	Tetracycline	NT	NA	4	S
	Trimethoprim/sulfamethoxazole	<2/38	S	<0.5/9.5	S
	Meropenem	NT	NA	1	‡
2, sputum culture	Amoxicillin/clavulanate	NT	NA	4/2	S
	Ceftazidime	4	S	4	S
	Doxycycline	NT	NA	1	S
	Imipenem	NT	NA	0.5	S
	Tetracycline	NT	NA	4	S
	Trimethoprim/sulfamethoxazole	<2/38	S	<0.5/9.5	S
	Meropenem	NT	NA	1	‡


*CDC, Centers for Disease Control and Prevention; NA, not applicable; NT, not tested; R, resistant; S, susceptible.  †Siemens Healthcare Diagnostics, https://www.siemens-healthineers.com. ‡There are no published breakpoints in the Clinical and Laboratory Standards Institute M45 (*9*).

Patient 1 returned to the ED 2 weeks later because of manifestations similar to those at the first visit. She was afebrile and admitted for diuresis. The left thigh lesion had progressed into a 2-cm, tender, shallow ulcer productive of purulent material surrounded by erythema and a focal area of induration (Figure). Laboratory data reflected a leukocyte count within reference ranges and mild renal insufficiency with estimated glomerular filtration rate of 40.47 mL/min (Table 2).

**Figure:**
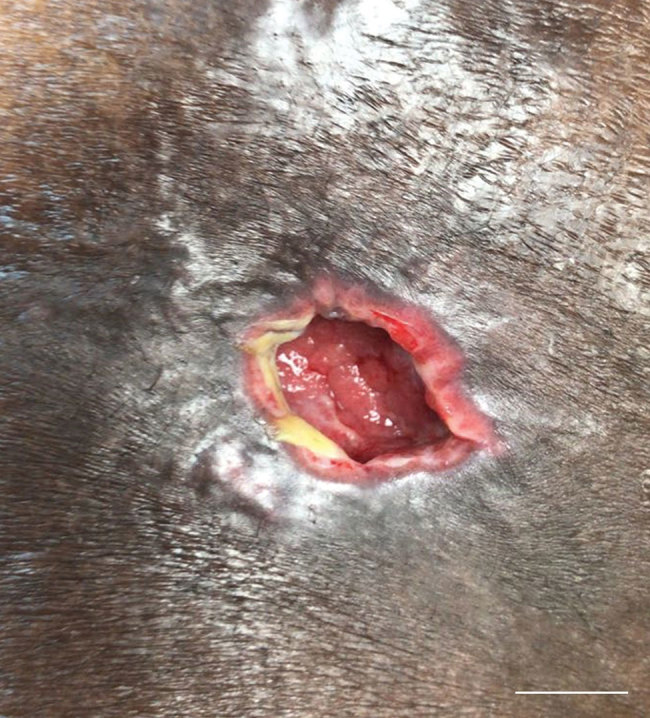
Cutaneous melioidosis lesion in case-patient 1 after Hurricanes Irma and Maria, St. Thomas/St. John District, US Virgin Islands, October 2017. This lesion was on the left anterior thigh and had a diameter of 2 cm. Shown is a tender, shallow, ulcer productive of purulent material surrounded by erythema and a focal area of induration. Scale bar indicates 1 cm.

**Table 2:** Laboratory values for 2 case-patients with melioidosis after Hurricanes Irma and Maria, St. Thomas/St. John District, US Virgin Islands, October 2017*

Parameter	Patient 1			Patient 2			Reference range
	Oct 18	Oct 21	Oct 24	Oct 26	Oct 27	Oct 30	
Leukocytes	4.2	NT	4.1	28.3	18.1	12.6	4.8–10.8 × 10^3^/mm^3^
Hemoglobin B	11.9	NT	15.5	11.3	10.4	8.1	12.0–14.0 g/L
Hematocrit	38.1	NT	48.9	34.6	31.5	24.0	36.0–42.0%
Platelets	185	NT	174	441	345	201	140–440 × 10^3^/mm^3^
Neutrophils	67.0	NT	46.2	92.5	89.5	92.6	40.0%–75.0%
Lymphocytes	20.8	NT	38.2	1.8	2.4	5.1	15.0%–45.5%
Monocytes	9.6	NT	10.8	4.9	5.3	2.2	0.0%–10.0%
Eosinophils	0.6	NT	3.7	0.7	2.7	0.0	0.0%–6.0%
Basophils	2.0	NT	1.1	0.1	0.1	0.1	0.0%–2.0%
Sodium	134	127	NT	125	130	137	136–145 mmol/L
Potassium	4.8	3.6	NT	3.5	2.9	3.2	3.6–5.2 mmol/L
Chloride	100	91	NT	87	95	104	98–107 mmol/L
Bicarbonate	28.0	31.3	NT	17.5	21.5	16.5	21–32 mmol/L
Blood urea nitrogen	23	18	NT	17	17	63	7–18 mg/dL
Creatinine	1.58	1.26	NT	1.19	1.07	3.92	0.6–1.3 mg/dL
Glucose	169	213	NT	367	235	404	70–110 mg/dL
Hemoglobin A1C	NT	NT	NT	NT	NT	11	4.5%–6.2%
Calcium	8.2	9.1	NT	10.2	9.0	8.3	8.5–10.5 mg/dL
Phosphorus	NT	3.3	NT	NT	NT	1.9	2.4–4.9 mg/dL
Magnesium	NT	NT	NT	NT	NT	1.9	1.8–2.4 mg/dL
Total bilirubin	0.6	NT	NT	1.5	1.5	2.3	0.0–1.0 mg/dL
Direct bilirubin	NT	NT	NT	NT	NT	2.0	0.0–0.3 md/dL
AST	32	NT	NT	34	52	49	15–37 U/L
ALT	27	NT	NT	25	25	34	12–78 U/L
Alkaline phosphatase	94	NT	NT	155	138	142	50–136 U/L
Total protein	7.2	NT	NT	8.0	6.4	5.1	6.4–8.2 g/dL
Albumin	3.10	2.70	NT	2.10	1.6	0.8	3.4–5.0 g/dL

*Units for laboratory values are shown in the reference range column. ALT, alanine aminotransferase; AST, aspartate aminotransferase; hemoglobin A1C, glycated hemoglobin; NT, not tested.

A second wound culture was collected, and the patient was given intravenous meropenem (1 g every 8 h). Culture was presumptively positive for *B. pseudomallei* and *Serratia marcescens* after ≈48 hours, confirmed after 8 days. Both isolates showed the same resistance pattern and were susceptible to meropenem and trimethoprim/sulfamethoxazole: the MIC for meropenem was <1 µg/mL (Table 2). Meropenem was continued for 8 days, and ulcer improvement was observed. The patient was discharged while receiving oral trimethoprim/sulfamethoxazole (800 mg/160 mg 2×/d) to complete maintenance therapy. The patient completed a 3-month course of trimethoprim/sulfamethoxazole and achieved resolution.

Patient 2 was a 60-year-old female who was a resident of St. John who had diabetes. She was referred to the ED at Schneider Regional Medical Center by her primary care physician because of hyperglycemia, productive cough, and malaise for 1 week (symptom onset 46 days after Hurricane Irma and 33 days after Hurricane Maria). The patient was admitted to the intensive care unit because of community-acquired pneumonia.

The patient was lethargic and had a temperature of 101°F; heart rate was 99 beats/min, respiratory rate 22 breaths/min, and blood pressure 142/81 mm Hg. Blood gas testing showed pO2 of 47.6 mm Hg with an oxygen saturation of 87.2% on 2-liter nasal cannula. A chest radiograph showed a left-sided mild infiltrate, and her leukocyte count was markedly increased (28.3 × 10^3^ cells/mm^3^) (Table 2).

The patient was given intravenous ceftriaxone (1 g/d) and azithromycin (500 mg/d) after blood and sputum cultures were prepared. She required bilevel positive airway pressure but eventually required mechanical ventilation. The patient then became hypotensive and required norepinephrine to maintain a main arterial pressure >65 mm Hg. Ceftriaxone was discontinued, and she was given intravenous piperacillin/tazobactam (3.375 g every 6 h). Trimethoprim/sulfamethoxazole- and ceftazidime-sensitive *B. pseudomallei* were identified from sputum culture after 72 hours (Table 1). Methicillin-sensitive *Staphylococcus aureus* and *Candida glabrata* were also identified. One of 2 blood cultures was positive for gram-negative rods. Piperacillin/tazobactam was discontinued, and the patient was given meropenem (1 g every 8 h).

The patient remained critically ill and was transferred to a tertiary care hospital in the continental United States. She died in a long-term care facility during October 2018 without showing signs of neurologic improvement.

Isolates from both patients showed susceptibility to routinely tested antimicrobial drugs (*10**,**11*). Isolates from patient 1 showed resistance to ceftazidime during preliminary analysis (Table 1). However, broth microdilution confirmatory testing performed at CDC indicated ceftazidime susceptibility, highlighting the need for additional antimicrobial resistance confirmation.

Both patients were interviewed to determine travel history and possible exposure sources. Patient 1 traveled occasionally to the southeastern United States; her last travel date was 3 months before her illness. This patient reported flooding and water damage to her home from the hurricanes, but did not report contact with flood waters. Patient 2 reported no travel history before the hurricanes.

VIDOH has investigated and confirmed a subsequent case-patient with pulmonary melioidosis in St. Thomas during December 2018 (I. Guendel et al., unpub. data). This case-patient reported no recent travel and might have had occupational exposure as a professional gardener. This person had 2 risk factors (type II diabetes mellitus and heavy use of alcohol).

## Conclusions

Given regional occurrence, detection of melioidosis in the US Virgin Islands is not surprising. Furthermore, emergence of melioidosis after extreme weather events has been well documented, and cases were likely acquired locally from storm-related exposure to flooded soil, surface water runoff, or generation of coarse aerosols (*12**,**13*). Although detection of *B. pseudomallei* has yet to be confirmed in the environment, it might be endemic to the US Virgin Islands, as in Puerto Rico.

In January 2018, melioidosis was listed as a reportable disease in the US Virgin Islands. Future actions include disease education efforts for physicians and laboratory staff because misdiagnosis is common (*14*). Awareness campaigns highlighting preventive measures for the public are necessary because risk factors are prevalent in the local population (e.g., diabetes and other chronic disease) and might be exacerbated under disaster settings (e.g., respiratory effects and open wounds). VIDOH has implemented rapid diagnostic testing by using Active Melioidosis Detect (InBios International, https://inbios.com) on suspected specimens for prompt on-island case identification while routine ED diagnostic cultures are performed (*5*). All confirmatory testing is conducted at CDC.

## References

[1] Wiersinga WJ, Currie BJ, Peacock SJ. Melioidosis. N Engl J Med. 2012;367:1035–44. 10.1056/NEJMra1204699 22970946

[2] Cheng AC, Currie BJ. Melioidosis: epidemiology, pathophysiology, and management. Clin Microbiol Rev. 2005;18:383–416. 10.1128/CMR.18.2.383-416.2005 15831829 PMC1082802

[3] Wuthiekanun V, Amornchai P, Saiprom N, Chantratita N, Chierakul W, Koh GC, et al. Survey of antimicrobial resistance in clinical *Burkholderia pseudomallei* isolates over two decades in Northeast Thailand. Antimicrob Agents Chemother. 2011;55:5388–91. 10.1128/AAC.05517-11 21876049 PMC3195054

[4] Stewart T, Engelthaler DM, Blaney DD, Tuanyok A, Wangsness E, Smith TL, et al. Epidemiology and investigation of melioidosis, Southern Arizona. Emerg Infect Dis. 2011;17:1286–8. 10.3201/eid1707.100661 21762589 PMC3381374

[5] Benoit TJ, Blaney DD, Doker TJ, Gee JE, Elrod MG, Rolim DB, et al. A review of melioidosis cases in the Americas. Am J Trop Med Hyg. 2015;93:1134–9. 10.4269/ajtmh.15-0405 26458779 PMC4674224

[6] Doker TJ, Sharp TM, Rivera-Garcia B, Perez-Padilla J, Benoit TJ, Ellis EM, et al. Contact investigation of melioidosis cases reveals regional endemicity in Puerto Rico. Clin Infect Dis. 2015;60:243–50. 10.1093/cid/ciu764 25270646

[7] Sanchez-Villamil JI, Torres AG. Melioidosis in Mexico, Central America, and the Caribbean. Trop Med Infect Dis. 2018;3:pii:24. 10.3390/tropicalmed3010024 PMC5958912 29780897

[8] Gee JE, Gulvik CA, Elrod MG, Batra D, Rowe LA, Sheth M, et al. Phylogeography of *Burkholderia pseudomallei* Isolates, Western Hemisphere. Emerg Infect Dis. 2017;23:1133–8. 10.3201/eid2307.161978 28628442 PMC5512505

[9] Clinical and Laboratory Standards Institute. Methods for antimicrobial dilution and disk susceptibility testing of infrequently isolated or fastidious bacteria. 3rd ed (M45). Wayne (PA): The Institute; 2016. 10.1086/510431 17173232

[10] Limmathurotsakul D, Peacock SJ. Melioidosis: a clinical overview. Br Med Bull. 2011;99:125–39. 10.1093/bmb/ldr007 21558159

[11] Wuthiekanun V, Peacock SJ. Management of melioidosis. Expert Rev Anti Infect Ther. 2006;4:445–55. 10.1586/14787210.4.3.445 16771621

[12] Cheng AC, Jacups SP, Gal D, Mayo M, Currie BJ. Extreme weather events and environmental contamination are associated with case-clusters of melioidosis in the Northern Territory of Australia. Int J Epidemiol. 2006;35:323–9. 10.1093/ije/dyi271 16326823

[13] Merritt AJ, Inglis TJJ. The role of climate in the epidemiology of melioidosis. Curr Trop Med Rep. 2017;4:185–91. 10.1007/s40475-017-0124-4 29188170 PMC5684260

[14] Hemarajata P, Baghdadi JD, Hoffman R, Humphries RM. *Burkholderia pseudomallei*: challenges for the clinical microbiology laboratory. J Clin Microbiol. 2016;54:2866–73. 10.1128/JCM.01636-16 27654336 PMC5121373

